# The Editor’s Choice for Issue 4, Volume 6

**DOI:** 10.3390/ijns7010014

**Published:** 2021-03-05

**Authors:** Peter C. J. I. Schielen

**Affiliations:** Office-International Society for Neonatal Screening, Reigerskamp 273, NL-3607 HP Maarssen, The Netherlands; peter.schielen@gmail.com

The Editorial team (Ralph Fingerhut, Can Ficiccioglu, Dianne Webster, David Millington and I) is trying something new. We will take turns in helping you to make your choice of the wide variety of papers we offer in each new issue of the *International Journal of Neonatal Screening* (*IJNS*). Here, I will kick this off, with an appraisal of Issue 4 of Volume 6 of *IJNS* [1].

Issue 4 lists 21 papers. Sadly, this issue holds two obituaries of our beloved colleagues Fred Lorey [2] (also on the cover of this issue) and Zoltan Lukacs [3], which you may want to read in their memory.

Then, many of these contributions belong to Special Issues; there are five papers in the Special Issue “Neonatal Screening for Mucopolysaccharidoses” [4], four in “Newborn Screening: Promoting Quality to Optimise Benefit and Reduce Harm” [5], three in “Economic Evaluations of Newborn Screening: Methodological Considerations and Applications” [6], two in “Newborn Screening for Pompe Disease” [7] and one in “CAH Screening—Challenges and Opportunities” [8]. I think that shows how successful we are with our Special Issues and how diverse we can be thematically.

The paper of Dr. Mei Baker and co-workers on molecular technologies in newborn screening is a must-read [9]. It is almost a “prequel” for an entire Special Issue on next generation sequencing in newborn screening that *IJNS* is preparing, and we already have some very exciting contributions to this Special Issue (hopefully to be published in the second half of 2021) [10].

Of course, we recommend you dive into all the papers of this issue, but my recommendation would be “We All Have a Role to Play: Redressing Inequities for Children Living with CAH and Other Chronic Health Conditions of Childhood in Resource-Poor Settings” by Kate Armstrong et al. [11]. Admittedly, this is a lengthy read, but it is worth it. The paper is at the heart of the mission of the International Society for Neonatal Screening (ISNS) to help all families and their babies through dissemination of knowledge. It connects all stakeholders in the supportive network around patients and supports the sharing of knowledge and building bridges between countries. The paper tells the story of what developed from the personal experience of the first author, who almost lost a three-week-old infant to an adrenal crisis, combined with her observation that children with CAH in Vietnam await a much darker fate as compared to Australian infants. This led her, in 2004, to found “Caring and Living as Neighbours (CLAN)”. CLAN is an Australian non-governmental organization committed to a rights-based approach to optimize the quality of life for children and young people living with CAH and other chronic health conditions in resource-poor settings. This paper is about equity. It systematically works towards that goal using the knowledge-to-action-framework. It also presents a second framework, the “five pillars” in short: (1) access to medicines and equipment, (2) access to education, research and advocacy, (3) optimization of medical management, (4) encouragement of family support groups, and (5) actions to reduce financial burdens and promote financial independence. The rest of the paper reads as a short novel, where step by step, systematically working from these frameworks, barriers are taken down to ultimately support the CAH community in Vietnam. Additionally, the paper presents the positive consequences of CLAN for Indonesia, the Philippines and Pakistan (and many other countries).

I feel that this paper is inspiring, with authors from five countries that together share a success story based on two frameworks. These frameworks can easily be adopted and applied in other regional settings. In the words of the authors: “share the wheel, don’t reinvent it”. This paper is about promoting equity and disseminating knowledge, and that, I repeat, is at the heart of the mission of ISNS.
